# Filled Pause Refinement Based on the Pronunciation Probability for Lecture Speech

**DOI:** 10.1371/journal.pone.0123466

**Published:** 2015-04-10

**Authors:** Yan-Hua Long, Hong Ye

**Affiliations:** Department of Electronical and Information Engineering, Shanghai Normal University, Shanghai, China; The University of Science and Technology of China, CHINA

## Abstract

Nowadays, although automatic speech recognition has become quite proficient in recognizing or transcribing well-prepared fluent speech, the transcription of speech that contains many disfluencies remains problematic, such as spontaneous conversational and lecture speech. Filled pauses (FPs) are the most frequently occurring disfluencies in this type of speech. Most recent studies have shown that FPs are widely believed to increase the error rates for state-of-the-art speech transcription, primarily because most FPs are not well annotated or provided in training data transcriptions and because of the similarities in acoustic characteristics between FPs and some common non-content words. To enhance the speech transcription system, we propose a new automatic refinement approach to detect FPs in British English lecture speech transcription. This approach combines the pronunciation probabilities for each word in the dictionary and acoustic language model scores for FP refinement through a modified speech recognition forced-alignment framework. We evaluate the proposed approach on the Reith Lectures speech transcription task, in which only imperfect training transcriptions are available. Successful results are achieved for both the development and evaluation datasets. Acoustic models trained on different styles of speech genres have been investigated with respect to FP refinement. To further validate the effectiveness of the proposed approach, speech transcription performance has also been examined using systems built on training data transcriptions with and without FP refinement.

## Introduction

Speech disfluencies are common phenomena in spontaneous and lecture speech (e.g., filled pauses, repetitions, and repairs) [1]. In most lecture speech, the most frequently occurring disfluencies are filled pauses (FPs), especially when the topic is unfamiliar and when speakers are uncertain or need to make decisions. FPs are an integral part of how human speak, can provide valuable information about the speaker’s cognitive state, and can be critical for successful turn-taking [2]. However, for automatic speech transcription systems, FPs have been shown to be problematic because they can be confused with and recognized as small functional words, usually resulting in fragment-like structures that increase transcription error rates [3–6]. Consideration of how to handle FPs is indispensable to the development of robust speech transcription systems. Actually, the detection of FPs not only can improve the performance of speech transcription systems, but also helps to enhance speech synthesis systems [7, 8], to address the plural idiosyncrasies of speakers, etc [9–11].

There has been a great deal of research devoted to automatic disfluency detection, and FPs are likely the most studied type of disfluency [1, 4–6, 12]. A log-linear reranker noisy channel model was proposed by [12] to repair the disfluency detection. In [13], FPs in conversational speech were detected by inserting optional FPs between any two words and at the beginning and end of each utterance lattice during decoding. In [1, 14–17], the researchers were focused on extracting effective features, such as prosodic, formant, syllable and acoustic features or a combination of them. Meanwhile, different methods have also been proposed to provide good generative or discriminative disfluency models to detect FPs, such as CRFs (Conditional Random Fields) [18], CARTs (Classification and Regression Trees) [19], and ILP (Integer Linear Programming) [20]. To some degree, the abovementioned detection approaches have already achieved successful results, with most of them being primarily based on training data with accurate manual disfluency transcriptions of speech and textual information.

In this study, we investigate what happens to a speech transcription system when we move to a new domain containing no in-domain data annotated with disfluencies to be used for training. Such a situation arises when we begin developing a transcription system for a new task. In contrast with the abovementioned FP detection task, we concentrate on addressing the FP refinement issue for lecture speech transcription, in which there are only a few imperfect transcriptions of training data available. We use “FP refinement” rather than “FP detection” in this paper because our goal is both to detect FPs and to refine the full quality of speech transcription to improve the ultimate development of speech transcription systems.

It is well known that annotating FPs present in the audio signal by a human expert is a time-consuming and expensive task because of their perceptually ambiguous distinction characteristics, particularly for current speech transcription systems that use a large amount of data for training. Therefore, an automatic framework to refine FPs in the available training data transcriptions is necessary. Unlike the previous works on using complicate features or search methods, this paper investigates a new approach by using a modified forced alignment procedure to identify potential existing FPs in speech. For each normal word in the dictionary, pronunciations of possible FPs and their corresponding pronunciation probabilities are properly assigned for alignment. Moreover, some unreasonably short FP insertions are filtered out as a post-processing step of the refinement.

The performance of the proposed approach was evaluated using a British Reith Lectures dataset. Experimental results for both the development and evaluation datasets have verified the effectiveness and great success of the proposed approach. Furthermore, the results show that the FPs in training speech data are well refined and that speech transcription performance also significantly improves when comparing systems trained on transcriptions with and without FP refinement.

This paper is organized as follows. Our proposed automatic FP refinement approach is first introduced in detail. The experimental setup and results are then presented and analyzed, followed by the conclusion and future work.

## Automatic Filled Pause Refinement

In this section, we propose an approach to automatically detect FPs given speech transcripts that omit FPs but are otherwise accurate. This approach is motivated by situations in which only a “cleaned-up” transcript is available, while an accurate verbatim transcript is to be recovered automatically. We treat this issue as an FP refinement problem, and we investigate how effectively it is solved by a large, state-of-the-art continuous-speech vocabulary transcription system.

**Fig 1:**
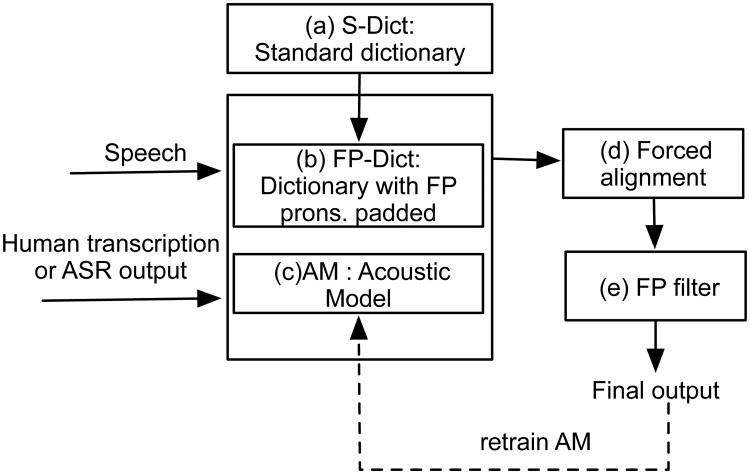
Framework of the automatic filled pause refinement.

Fig 1 illustrates the proposed framework for automatic FP refinement. Given speech segments and their corresponding human transcriptions or automatic speech recognition (ASR) outputs, a pre-trained acoustic model (AM) and a well-prepared dictionary (FP-Dict in Fig 1) are then used to conduct a forced alignment to identify potential FPs in the audio data. A filter is further designed as a post-processing step to remove unreasonable short FPs before achieving the final output. Actually, to transcribe new-domain speech data, the AMs used for forced alignment are usually trained on out-of-domain data. To avoid biasing the FP refinement with poor results obtained with an out-of-domain recognizer, after an initial FP refinement of the training data transcriptions, the in-domain training data may be further added to out-of-domain data to retrain the AMs or may be directly used to update the old in-domain acoustic models to enhance refinement performance in 2 ∼ 3 iterations. Hence, the proposed approach can be regarded as searching the pronunciation knowledge of each word in dictionary and acoustic model knowledge sources in a search framework for FP detection. This framework is similar to the traditional forced alignment procedure in ASR, except blocks (b) and (e) in Fig 1.

### Principle to Prepare FP-Dict

The principle to obtain the dictionary “FP-Dict” in Fig 1 is presented in this subsection. Given a speech segment *X* and its corresponding transcription, the aim of forced alignment is to map the transcribed words into a phone sequence using a pronunciation dictionary. The state-of-the-art Hidden Markov Model (HMM)-based speech recognition or transcription forced alignment is intended to align the frames with phonemes via the Viterbi algorithm, which finds the phoneme sequence $\hat{\text{p}}$ with the start time that has the largest posterior probability *P*(*p*∣**X**) given the observed data and the acoustic model represented by the HMMs as the objective function in Eq (1):

$$\begin{array}{c} \hat{p}=\underset{p}{\arg\max}P\left(p|X\right)=\underset{p}{\arg\max}P\left(X|p\right)P\left(p\right) \end{array}$$ (1)

where the term *P*(**X**∣*p*) is the acoustic score and *P*(*p*) is the language model score during Viterbi alignment based on the HTK Speech Recognition Toolkit [21].

**Fig 2:**
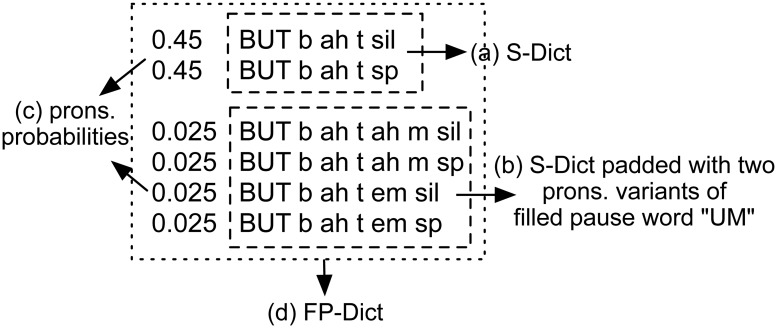
FP-Dict illustration of word “BUT”.

During standard forced alignment, the term *P*(*p*) can be disregarded because it is constant when the transcription and dictionary are fixed. Therefore, the most likely phone sequence path in Viterbi search is decided only by acoustic scores, which varies with different pronunciation variants of each word given in the dictionary. Therefore, we propose increasing the probability of the Viterbi search identifying potential phone sequences of FPs by adding new pronunciation variants to each word in the dictionary, as illustrated in Fig 2. Furthermore, to control for the degree of FPs, a probability is assigned to each pronunciation variant of the word according to Eq (2) and Eq (3).

$$\begin{array}{ccc} \sum_{i=1}^{M}P\left(fp_{i}|W\right) & = & P_{fp}\\ \sum_{j=1}^{N}P\left(std_{j}|W\right) & = & P_{std} \end{array}$$ (2)

satisfying

$$\begin{array}{c} P_{fp}+P_{std}=1 \end{array}$$ (3)

where *P*(*fp*_*i*_∣*W*) and *P*(*std*_*j*_∣*W*) are the pronunciation probabilities for word “W” with FP pronunciation padded and the standard pronunciation, respectively.

As illustrated in Fig 2, a proposed FP-Dict for the word “BUT” is composed of the S-Dict (block (a)), which contains the standard pronunciation of “BUT”, and the S-Dict padded with two pronunciation variants of the FP “UM”. In addition, we assign each part of the *M* or *N* pronunciations of the word “BUT” with zero knowledge prior to probability *P*_*fp*_/*M* or *P*_*std*_/*N*.

It is obvious that once the word list of FPs is given, the proposed FP-Dict is then fixed. Given the human transcriptions or ASR outputs of the audio data, when we perform forced alignment according to Eq (1), if the acoustic score of a word phone sequence with standard pronunciation is lower than the one with the same phone sequence but with FP pronunciation padded, then we can conclude that a FP exists between the given current word and the next word. Furthermore, a word pronunciation probability ratio *W*_*ppr*_ is used to flexibly control the possibilities of FP words appearing.

$$\begin{array}{c} W_{ppr}=\frac{P_{fp}}{P_{std}}={\left(W_{ppr}^{base}\right)}^{r} \end{array}$$ (4)

where $W_{ppr}^{base}$ refers to a basic word pronunciation probability ratio when we prepare the FP-Dict. To avoid modifying the FP-Dict when a new *W*_*ppr*_ is used, the scale factor *r* is introduced. Therefore, once the FP-Dict has been fixed, it is easy to obtain a FP-Dict with a new *W*_*ppr*_ by merely changing *r* rather than modifying all of the numbers of pronunciation probabilities. Eq (4) clearly shows that if we decrease *r*, then there is a greater likelihood of identifying FP words during forced alignment; otherwise, larger *r* indicates a greater likelihood of aligning the speech with the normal sequence of phones.

### FP Filter Design

Silence, breath or background noise can easily become misaligned and confused with FPs because of their similar acoustic characteristics, especially in conversational speech. This is the main contributor to insertion errors in state-of-the-art speech recognition and transcription systems. Therefore, to remove unreasonable short FPs refined from forced alignment, using our proposed FP-Dict in the previous section, a FP filter is designed as a post-processing step in the automatic FP refinement framework in the following heuristic way:

$$\begin{array}{c} L_{fp}=\#frames\;of\;aligned\;filled\;pause \end{array}$$ (5)

where *L*_*fp*_ refers to the number of aligned frames of FPs refined using FP-Dict. When the *L*_*fp*_ is larger than a certain threshold, these refined FPs are retained in the aligned transcription; otherwise, they are stripped off. The threshold is normally set to 6 if the Hidden Markov Model has 5 states for each Gaussian mixture.

### Refinement Procedure

According to Fig 1, regarding automatically refined FPs for a new corpus (which could also reflect a change in transcription tasks), our proposed procedure entails the following sequence of operations once the audio data and human transcription or ASR output files have been loaded:

- **Step 1**: Prepare well-trained acoustic models (AMs).
- **Step 2**: Prepare a standard dictionary (S-Dict in Fig 1) that covers all of the words included in the given transcriptions to be refined.
- **Step 3**: Produce a word list of filled pauses (FP list) that were detected, and prepare the pronunciation variants for each word in the FP list.
- **Step 4**: Construct the FP-Dict according to the principle described in the previous section.
- **Step 5**: Perform Viterbi forced alignment on all the audio data for which the transcriptions need to be refined, using FP-Dict and the prepared AMs in Step 1.
- **Step 6**: Filter short FPs to reduce unreasonable insertions as described in the above section.
- **Step 7**: If needed, retrain the in-domain acoustic models using the successful aligned data with the refined transcriptions derived from the output of (e) step in Fig 1, or retrain the out-of-domain acoustic models by adding the FP refined data into training. Then, repeat from step 6.

Please note that there is a minor engineering implementation trick when we refine the FPs at word level after Step 5: when we construct the FP-Dict in Step 4, we also produce a projection file at the same time, the projection file indicates word projections between normal words and words padded with FPs. After performing forced alignment using FP-Dict, the outputs can be both of the aligned words and their corresponding phone sequences, then the FPs can be recovered by doing a word projection according to the forced alignment outputs and the projection file. For instance, given the example in Fig 2, if the aligned phone sequence is “b ah t em sp”, then a word projection “BUT UM = BUT b ah t em sp” must be found in the projection file. Then we can decide the recovered word should be two words “BUT UM” instead of one word “BUT”.

By using the above proposed refinement procedure, we can refine the FPs automatically instead of manually annotating them by human experts. This can save a lot of time and money.

The question of how to define a suitable FP list to construct the FP-Dict is highly dependent on the speech transcription task. For example, for spontaneous English speech, *uh* and *um* must be included in this list, whereas for a Mandarin task, the speakers intensively use *zhege (literally ‘this’)* and *nage (literally ‘that’)* and *uh/mm* as FPs [13]. In general, this list is generated manually according to prior domain knowledge about transcription tasks. Another method is to automatically collect FPs from the lightly supervised decoding output of all audio data, particularly when only non-labelled or imperfect transcriptions are available.

In addition to the FP-Dict, the acoustic model also plays an important role in successfully refining FPs. Normally, to force-align a new corpus, the AM used in step 1) is well trained using the in-domain data in which the transcriptions are accurately annotated. Acceptable refinement performance can be achieved under this situation. Therefore, there is no need to retrain the AM after FP filtering. These refined transcriptions can be directly used to train the speech transcription and recognition systems. However, if the new corpus domain is different from the AM training data for alignment, then it is obvious that the quality of the retrained AM can be greatly improved by adding the successfully aligned in-domain data. Additionally, in most state-of-the-art speech transcription tasks, only imperfect transcripts are available; lightly supervised training is often used to obtain better transcripts to train acoustic models. In this case, we can also use the proposed approach to directly refine FPs to achieve better or more accurate transcripts. Furthermore, once we have a sufficiently large speech corpus with FP transcriptions available, we can create dedicated acoustic FP models to supplement the traditional triphone model set to improve speech transcription system building.

## Experimental Setup

### Database

The British Broadcasting Corporation (BBC) has opened most of the content of its broadcast archives to the public. The Reith Lectures is one of the archives we use to evaluate the proposed FP refinement approach in this paper. The Reith Lectures is a series of annual radio lectures on significant contemporary issues given by leading figures from relevant fields. A total of 160 episodes are included in this work, covering the years from 1980 to 2011. One leading figure was invited to deliver a range of 3–6 episodes at different times on the radio each year, with the exception of several special years. We manually labelled each episode with two regions: a lecture region given by the leading speaker and a non-lecture region that contained the introduction of the given lecture by the presenter Sue Lawley. Moreover, a question & answer session has been followed by the main lecture since 1988. Only the main lecture regions of all audio data were used for the evaluation and system building. Episode durations range from 20 to 60 minutes, yielding a total duration of 76 hours. We divided the data at the episode level into a training set of 68.2 hours (bbc.train), a development set of 4.1 hours (bbc.dev) and a test set of 3.7 hours (bbc.eval). The FP rate (the ratio of the number of FPs to the total number of words in the reference) for bbc.dev and bbc.eval are approximately 1.6% and 2.3%.

All of the audio data and lecture transcripts of these lectures can be downloaded for free from the BBC website [22]. However, the public released transcripts are not verbatim transcripts, as they contain a number of errors (substitutions, deletions and insertions), depending on the degree to which the speaker deviated from his/her prepared scripts before the speech. Meanwhile, no time stamps are included in these released transcriptions. Therefore, before refining the FPs using the proposed approach, we first refined the transcriptions and time stamps using a lightly supervised approach based on decoding with a biased language model [23]. Details of this light refinement approach are reported in the LST system description part in the 4th subsection (Transcription Systems). In addition, the transcriptions of bbc.dev and bbc.eval were manually corrected using a transcription tool XTrans (available from: https://www.ldc.upenn.edu/language-resources/tools/xtrans/downloads) to produce gold-standard transcriptions (including accurate FPs) for system evaluation.

### Performance Measurement

To evaluate the performance of the FP refinement approach, four standard metrics proposed in the literature are used. The first metric is Precision (Prec.), which is the ratio of the correctly refined tags X to all of the detected tags X by the proposed FP refinement system (where X is FP). The second metric is Recall (Rec.), which is the ratio of correctly refined tags X to all of the tags X that appear in the gold-standard reference transcriptions. The third metric is the DET curves [24], which are used to represent the range of possible system operating points of refinement systems. The fourth metric is the pair of false alarm (incorrect refined, FA) and missed alarm (failed refined, MA) probabilities that are used to plot the DET curve. Note that the false alarm can be greater than 100%. See the following example:

- REF: ** they think er ** they don’t
- HYP: UH they think er UM they don’t
- Eval: I     I

where two insertion errors for the FP word “uh” and “um” result in a false alarm of 200% because there is only one FP word “er” in the reference.

We also compute the classical NIST (National Institute of Standards and Technology) Word Error Rate (WER), which is the sum error rate of insertions (Ins), deletions (Del) and substitutions (Sub) as the performance measure for transcription systems. Please note that when evaluating speech transcription systems, FPs appearing in reference are removed, meaning that the WERs only measure errors related to normal words.

### FP-Dict Construction

All of the word pronunciations used in this work are derived from the BEEP dictionary [25], which has phonemic transcriptions for more than 250,000 English words with British English pronunciations. The same is used for the standard dictionary (S-Dict in Fig 1) used for the FP refinement of BBC Reith Lectures. For those words that cannot be found in the BEEP, we used Sequitur G2P (grapheme-to-phoneme) [26] to generate pronunciations from BEEP automatically.

The word list of FPs that we considered to be refined in lectures was defined by analyzing the frequency of appearances of FPs in the reference transcriptions of bbc.dev. We verified that FPs occur primarily in prepositions and in the first syllable as well as before or after a sentence. Sometimes the difference between FPs is not obvious, and perceptually, their distinction is also ambiguous without a context, even for speech scientists. Therefore, in this study, those FPs that have nearly the same acoustic characteristics were merged together, whereas those with very few occurrences were ignored. Only the six most frequent FPs listed below were taken to be refined.

Filled Pauses: um, uh, er, ah, ha, huh

Given the S-Dict and FP word list, the FP-Dict was constructed by padding the pronunciations (multiple variants) of the six FPs above and adding pronunciation probabilities to each of the words in S-Dict according to the principle described in the previous section (illustrated in Fig 2). In addition, the basic word pronunciation probability ratio $W_{ppr}^{base}=1/9$, which means that a very small percentage of FPs in lecture speech is allowed. During experiments, it is easy to control the degree of FP refinement simply by changing the scale factor *r*.

### FP Refinement System

Three systems using the same dictionary (FP-Dict) but with different acoustic models were built to refine the FPs. Investigative experiments were performed with bbc.dev and bbc.eval, in which all annotated FPs in the reference transcriptions were removed; then, these transcriptions were used to perform the FP refinement for evaluating the proposed approach. There were no AMs trained on the in-domain data before the Reith Lectures available. The first system (termed SWB-FP) used AMs that were trained with approximately 62 hours of English conversational telephone speech taken from the Switchboard-I corpus (SWB) [27]. SWB is a corpus traditionally used for speech disfluency experiments because of the large amount of manually annotated disfluencies that it contains.

The 1997 English Broadcast News (BN) HUB4 [28] Speech corpus was used for the second system (termed BN-FP) building. Approximately 70 hours of data were obtained from HUB4 for acoustic model training, with nearly the same amount of data as in the SWB-FP and RL-FP (described below) systems. SWB and BN are very different genres. Speech in BN contains fewer FPs, sentences are longer and more grammatical, and the speakers are primarily professionals reading teleprompted text. By contrast, speech in SWB is more conversational, containing many backchannels and filler words.

The third system (termed RL-FP) used AMs trained with the same 60.2 hours of successfully aligned matching segments of BBC lecture data bbc.train as used for the LST system training described in the next section.

The architecture of AM training for these three systems is standard in modern, state-of-the-art speech transcription systems. In addition, 13-dimensional perceptual linear prediction coefficients (PLPs) and as high as third-order temporal derivatives were extracted with standard cepstral mean and variance normalization at the segment level. These PLPs were then reduced to 39 dimensions using heteroskedastic linear discriminant analysis (HLDA) [29]. Cross-word triphone GMM-HMM acoustic models were trained using ML estimation and were updated discriminatively using variants of minimum phone error (MPE) training with maximum mutual information (MMI) priors [30]. Models were tied with phonetic decision trees to yield a total of approximately 4,000 tied states with an average of 16 mixture components per state.

### Transcription Systems

The aim of the proposed FP refinement approach is to improve real speech transcription systems even when there are only imperfect transcriptions with no FPs annotated for training data available. Two Reith Lecture speech transcription systems, termed LST and LST-FP, were built using only the in-domain lecture data to further evaluate the proposed approach. Performance was also examined with the bbc.dev and bbc.eval datasets.

For ease of comparison and to underline the contributions of different AMs, the same dictionary, the trigram language model (LM), has been used. This LM was built by linearly interpolating a trigram LM estimated for the SWB [27] and a model trained on the in-domain BBC Reith lecture training transcriptions. The in-domain BBC LM has an interpolation weight of 0.73. The SRILM toolkit [31] and Kneser-Ney smoothing [32] were applied for LM estimation. The final interpolated LM contains 62K word unigrams with a perplexity of 124 and 117 and out-of-vocabulary (OOV) rates of 0.92% and 0.89% for the bbc.dev and bbc.eval, respectively. A two-pass decoding architecture was used for the decoding of two systems: the first pass generated initial transcriptions using unadapted models, and the second pass used speaker-adapted models to produce the final transcription.

**LST**. Because the publicly released raw transcriptions of Reith Lectures are imperfect and because no time stamps are available, a lightly supervised decoding was used to first refine the transcriptions automatically [23]. Each episode was first automatically segmented and clustered by speaker using an STT-based system similar to [33]. Each speech segment was then decoded in two passes using speaker adaptation, with the decoding employing a biased language model trained on all of the raw Reith Lecture transcriptions. A word-level transcription combination technique was used during lightly supervised decoding to obtain more accurate transcriptions [34]. The decoder output was compared with the raw transcription to identify matching sequences. Non-matching word sequences from the raw transcription were force-aligned with the remaining speech segments. Once realigned, the position of time stamps in the transcription could be corrected [35]. Furthermore, to achieve maximal training data, those segments with the same forced-aligned phone sequences of the decoder outputs as the raw transcriptions were selected from the bbc.train to train AMs of the LST speech transcription system. After this selection, only 60.2 hours of lecture speech data were ultimately retained to train the LST speech transcription system.

**LST-FP**. The LST-FP transcription system differs from the system LST in the amount of data and the quality of transcription of the AM training data. To obtain AM training data for this system, two cases must be considered: (1) For the 60.2 hours of segments with matched phone sequences already selected for LST system training, the proposed FP refinement was performed directly on the outputs of lightly supervised decoding. Additionally, the refined outputs from decoder were used as the final transcription for AM training in LST-FP. (2) For those segments with non-matching phone sequences, instead of discarding them as we did for LST training data selection, the FP refinement was performed on the lightly supervised decoding outputs before data selection. After FP refinement, we then force-aligned the decoder outputs with FP-refined and raw transcriptions provided by BBC to further select the segments with matching phone sequences for LST-FP AM training. The system used for both two cases of FP refinement was the RL-FP system. Therefore, after considering the two cases, we supplemented the 60.2 hours of data with 64 hours of transcriptions improved by FP refinement for LST-FP system training.

For both the LST and LST-FP speech transcription system building, the same AM training architecture was used, as presented in the above section on building FP refinement systems.

## Results and Discussion

### Performance of Lightly Supervised Decoding

To examine the quality of transcriptions derived from the lightly supervised decoding system with AMs trained on different speech genres, Table 1 presents the results for the bbc.dev dataset using SWB-FP.AM and BN-FP.AM, which were the same AMs used in the SWB-FP and BN-FP systems, respectively.

**Table 1:** Performance (in %) comparison between two lightly supervised decoding systems using different acoustic models on bbc.dev.

**Acoustic Model**	Sub	Del	Ins	**WER**
SWB-FP.AM	6.0	2.4	2.0	10.4
BN-FP.AM	6.1	2.6	2.3	11.0

Table 1 clearly shows that the AM trained on conversational speech data, SWB, achieved much lower error rates than the model trained on Broadcast News, especially in deletion and insertion errors. After a deep analysis of those deleted and inserted words, we found that the increased deletions and insertions produced by BN-FP.AM primarily derive from the confusion between FPs and other normal words. The large amount of manually annotated disfluencies in the SWB corpus resulted in better FP models than the BN data.

Furthermore, we also found a high-frequency substitution error resulting from the FPs that are easily confused in acoustic models because of the similar acoustic characteristics between the FPs and non-FP words. For the 6.0% and 6.1% substitutions in Table 1, 0.72% and 0.86% were due to the FPs. All of the above findings emphasize the importance of refining FPs in the training data transcriptions for a speech transcription system.

### FP Refinement Results

In this section, we present the experimental results in evaluating the effectiveness of the proposed FP refinement systems.

**Scale Factor Investigation**. As discussed in the previous section, instead of modifying all of the numbers of pronunciation probabilities, we can easily obtain a FP-Dict with new *W*_*ppr*_ by simply changing the scale factor *r* of the basic pronunciation probability ratio $W_{ppr}^{base}$. To find an optimal configuration, both the SWB-FP and BN-FP systems were tested.

**Fig 3:**
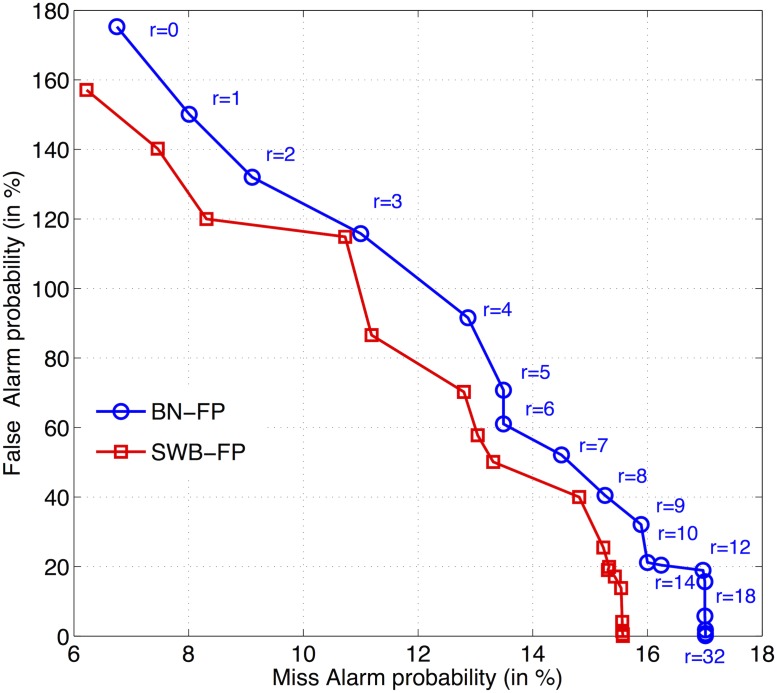
DET curves comparison of FP refinement performances varying with the scale factor *r* of pronunciation probability ratio.

Given $W_{ppr}^{base}=1/9$, the FP refinement results with the scale factor *r* changing from 0 to 32 on bbc.dev are shown in Fig 3. Two findings emerge from the DET curves: (1) Given the same fixed scale factors, most of the false and missed-alarm probabilities obtained by SWB-FP system are smaller than those obtained by the BN-FP system. This result implies that the FP acoustic models are better trained in the SWB-FP system than in the BN-FP system, and the AMs of SWB-FP may result in fewer insertion and deletion errors for a speech transcription task that contains many disfluencies. (2) Larger values of *r* are associated with fewer false alarms but more missed alarms. Hence, a larger scale factor leads to smaller word pronunciation probability ratios, which indicates less probability of identifying FP words during forced alignment. Most importantly, we can clearly observe that both of the two systems achieved the best results when *r* > = 14, and the FAs and MAs converged to approximately 0.0 and 15% for the SWB-FP system and 17% for the BN-FP system with an increase in *r*. Therefore, *r* = 14 was used for all of the following FP refinement systems.

**Performance of Three FP Refinement Systems**. Table 2 shows the performance comparison of three different FP refinement systems for both the bbc.dev and bbc.eval datasets. Please note that we did not retrain the acoustic models to perform the refinement as proposed in Fig 1, as both of the transcriptions from SWB and BN are accurate. Therefore, to make the results comparable, all of the results reported in this table were derived from the first (iteration 1) output from the FP filter.

**Table 2:** Comparison of Performance (in %) among three different FP refinement systems on bbc.dev and bbc.eval.

	bbc.dev				bbc.eval			
**System**	**Prec.**	**Rec.**	FA	MA	**Prec.**	**Rec.**	FA	MA
SWB-FP	86.0	84.5	13.8	15.5	87.0	87.2	13.0	12.8
BN-FP	84.2	83.0	15.6	17.0	83.2	83.8	16.9	16.2
RL-FP	83.8	83.3	16.1	16.7	84.7	85.0	15.4	15.0

Table 2 clearly shows that for the bbc.dev, the BN-FP and RL-FP systems achieved nearly the same results; however, much better results were obtained by the RL-FP system on the bbc.eval dataset than the BN-FP system did, which indicates that the acoustic properties of the development and evaluation datasets are less similar than we expected. Notably, the performance gaps between these two systems and the SWB-FP system are very large for both bbc.dev and bbc.eval. For example, using bbc.dev, by comparing the BN-FP with the SWB-FP system, the false and missed alarms were reduced from 15.6% to 13.8% and from 17.0% to 15.5%, respectively, which resulted in 1.8% and 1.5% absolute improvements in Precision and Recall. Meanwhile, it is clear that larger error reductions or performance improvements have been achieved for bbc.eval, and similar observations can be made by comparing SWB-FP with the RL-FP system. As shown in Table 1, these results also imply that the FP rate in the transcriptions of AM training for both the Broadcast News and Reith Lectures are very similar and much lower than that for Switchboard conversational speech. This result is observed because of the reading nature of news broadcasting and the non-verbatim prepared scripts of lectures.

Most importantly, all of the results in Table 2 demonstrate that our proposed FP refinement approach works well, even under a situation with few or no FPs available in the training data transcriptions. This finding is extremely important for when we move to a new domain containing no in-domain data annotated with FPs to be used in transcription system training.

**Acoustic Model Updating Investigation**. Note the validation FP refinement experiments for bbc.dev and bbc.eval in Table 2. Table 3 demonstrates the effectiveness of our proposed approach by updating the AMs of the RL-FP system iteratively, as shown in blocks (e) to (c) in Fig 1. To facilitate comparison, we listed the results on bbc.dev for the RL-FP system in Table 2 as the results for iteration 1, in which the FP acoustic events were not annotated or provided for AM training. For the second iteration, we first run the RL-FP system to refine the FPs for all of its training data, then use these refined transcriptions to retrain the AMs, and finally use the retrained AMs to refine the FPs in bbc.dev. Based on iteration 2, a third iteration was also implemented.

**Table 3:** Performance comparison on bbc.dev between RL-FP systems with different iteration of Acoustic Model updating.

#iteration	FA	MA
1	16.1	16.7
2	11.6	10.2
3	7.2	4.1

As shown in Table 3, absolute rates of 4.5% false alarms and 6.5% missed alarm error reduction have been achieved with the development dataset bbc.dev, based on a comparison of the results of iteration 1 with those of iteration 2. Further error reductions (4.4% of FA, 6.1% of MA) have been achieved by adding the third iteration to update the AMs for FP refinement. From these numbers, we can conclude that the performance improvements are significant when we retrain the acoustic models by adding the FPs that were refined in the previous iteration to supplement the training data transcription. Therefore, our proposed approach is very effective.

### Performance of Transcription Systems

To further explore the effectiveness of the proposed FP refinement approach, we now investigate how the refined FPs in AM training data affect a speech transcription system. Two speech transcription systems, LST and LST-FP, were built as described in the previous section. The speech transcription results for both the bbc.dev and bbc.eval datasets using these two systems are shown in Table 4.

**Table 4:** Speech transcription performances on bbc.dev and bbc.eval using system LST and LST-FP.

	bbc.dev		bbc.eval	
	LST	LST-FP	LST	LST-FP
Sub	15.1	14.6	16.0	15.1
Del	3.3	2.8	3.4	2.6
Ins	4.0	3.4	3.6	3.2
WER	22.4	20.8	23.0	20.9

Table 4 clearly shows that significant performance improvements have been achieved when comparing the results of the LST-FP system with those of the LST system, with a relative 7.1% and 9.1% WER reduction for bbc.dev and bbc.eval, respectively. These improvements are not reflected only in reductions of substitution errors; greater improvements are also found in the reduction of insertions and deletions. For bbc.dev, only a relative 3.3% substitution reduction can be achieved by the LST-FP system, but the relative deletion and insertion reductions are 15.2% and 15.0%, respectively. Meanwhile, for bbc.eval, these numbers are 5.6%, 23.5% and 11.1%, respectively. This result indicates that the proposed approach largely contributes to adequately addressing the acoustic confusion between FPs and those words that can be easily result in insertion and deletion errors during speech transcription, such as prepositions and conjunctions.

Therefore, we can conclude that all of the above observations clearly illustrate the importance of the sole difference between the LST and LST-FP systems: the transcription of FPs in Reith Lectures. The refined FPs not only result in more training data (approximately 4 hours) with matched phone sequences between lightly supervised decoding outputs and the raw imperfect transcriptions provided by BBC but also result in much better AMs for the LST-FP transcription system.

## Conclusion

In this paper, we have proposed a new FP refinement approach for speech transcription tasks. FPs can be refined through a modified forced alignment procedure by constructing a new dictionary with FP information integrated by assigning the proper pronunciation probability to each word. The proposed approach was evaluated using the Reith Lectures dataset provided by BBC. We found that increasing the word pronunciation probability ratio can decrease the likelihood of insertion errors appearing in FP detection. We also showed that it is important to use a high point in this approach (to avoid false alarms). Different acoustic models trained with different style of speech genres have been examined in performing the refinement. The performance of both the development and evaluation datasets demonstrated the effectiveness of the proposed approach. In addition, we observed that the iterative AM updating step of the proposed FP refinement framework is very useful when we develop a speech transcription system in a new domain that contains only imperfect transcriptions. Significant improvements have been obtained for the development dataset, thus verifying the necessity of AM updating.

Furthermore, our experiments building a lecture speech transcription system demonstrated that the recognition process can benefit greatly from the refinement of FP events prior to recognition. By further improving the FP detector, it should be possible to reduce the word error rate even further. Our investigation highlights the importance of continued research on disfluencies, such as FPs, to decrease recognition error rates in lecture speech. In addition, the computational complexity of our proposed approach is small because of the low need to use forced alignment. Therefore, the FP refined transcription can be obtained rapidly using the proposed FP refinement for state-of-the-art speech transcription systems that are usually trained on a very large amount of data. It is believed that this work is not the optimal approach to detect FPs, but it has proven to be useful for automatic or semi-automatic annotation.

In the future, several extensions may be worthwhile. First, all experiments in this paper are conducted using the lecture speech transcription task; thus, it is worth examining the effectiveness of even more challenging speech transcription or recognition tasks, such as spontaneous conversational speech and meeting speech. Second, the scale factor of the basic pronunciation probability ratio is determined using a development set; future research should explore other automatic methods. Third, many other studies can examine the FP detection issue, as further performance improvement may be achievable by fusing our proposed approach and other existing techniques, such as the techniques based on using prosodic features, or CRFs.
